# Heteromeric p97/p97^R155C^ Complexes Induce Dominant Negative Changes in Wild-Type and Autophagy 9-Deficient *Dictyostelium* strains

**DOI:** 10.1371/journal.pone.0046879

**Published:** 2012-10-03

**Authors:** Khalid Arhzaouy, Karl-Heinz Strucksberg, Sze Man Tung, Karthikeyan Tangavelou, Maria Stumpf, Jan Faix, Rolf Schröder, Christoph S. Clemen, Ludwig Eichinger

**Affiliations:** 1 Intitute for Biochemistry I, Medical Faculty, University of Cologne, Cologne, Germany; 2 Institute of Neuropathology, University Hospital Erlangen, Erlangen, Germany; 3 Institute for Biophysical Chemistry, Hannover Medical School, Hannover, Germany; Johns Hopkins School of Medicine, United States of America

## Abstract

Heterozygous mutations in the human VCP (p97) gene cause autosomal-dominant IBMPFD (inclusion body myopathy with early onset Paget’s disease of bone and frontotemporal dementia), ALS14 (amyotrophic lateral sclerosis with or without frontotemporal dementia) and HSP (hereditary spastic paraplegia). Most prevalent is the R155C point mutation. We studied the function of p97 in the social amoeba *Dictyostelium discoideum* and have generated strains that ectopically express wild-type (p97) or mutant p97 (p97^R155C^) fused to RFP in AX2 wild-type and autophagy 9 knock-out (ATG9^KO^) cells. Native gel electrophoresis showed that both p97 and p97^R155C^ assemble into hexamers. Co-immunoprecipitation studies revealed that endogenous p97 and p97^R155C^-RFP form heteromers. The mutant strains displayed changes in cell growth, phototaxis, development, proteasomal activity, ubiquitinylated proteins, and ATG8(LC3) indicating mis-regulation of multiple essential cellular processes. Additionally, immunofluorescence analysis revealed an increase of protein aggregates in ATG9^KO^/p97^R155C^-RFP and ATG9^KO^ cells. They were positive for ubiquitin in both strains, however, solely immunoreactive for p97 in the ATG9^KO^ mutant. A major finding is that the expression of p97^R155C^-RFP in the ATG9^KO^ strain partially or fully rescued the pleiotropic phenotype. We also observed dose-dependent effects of p97 on several cellular processes. Based on findings in the single versus the double mutants we propose a novel mode of p97 interaction with the core autophagy protein ATG9 which is based on mutual inhibition.

## Introduction

The late-onset autosomal dominant multisystem disorder IBMPFD is caused by mutations of the human p97 (synonyms: valosin containing protein (VCP) or TER ATPase in mammals, TER94 in *Caenorhabditis elegans*, Cdc48p in yeast, VAT in archaebacteria) gene on chromosome 9p13-p12 [1], [2]. At least 20 unique p97 missense mutations cause either IBMPFD [3]–[5], ALS14 [6], or HSP [7], [8] with codon 155 being a mutation hot spot. p97 is a ubiquitously expressed and evolutionarily highly conserved member of the AAA-ATPase family (ATPases Associated with a wide variety of cellular Activities). The protein has a tripartite structure comprising an N-terminal domain (CDC48) involved in ubiquitin binding, and two central D1 and D2 domains which bind and hydrolyze ATP [9]. p97 assembles into functional hexamers with the D domains forming a central cylinder, which is surrounded by the N-terminal domains [10]. In protein quality control and protein homeostasis p97 is a key player in endoplasmic reticulum associated protein degradation (ERAD), the ubiquitin proteasome protein degradation system (UPS), aggresome formation and autophagosome maturation [11]–[17].

Macroautophagy (hereafter autophagy) is an ancient cellular pathway to recycle cellular material that is conserved from yeast to man [18], [19]. More than 30 autophagy (ATG) genes have been identified, mainly in yeast, of which 18 constitute the core machinery for starvation induced autophagy. Autophagy contributes to many physiological and pathological processes, including cell differentiation and development, programmed cell death, cancer and neurodegenerative disorders [20].

The use of model organisms, such as *Saccharomyces cerevisiae*, *C. elegans*, *Drosophila melanogaster* or *D. discoideum*, in the study of the cellular consequences of mutations that cause human disease offers a number of advantages and has steadily increased in recent years. Disease-causing mutations can only be studied in a very limited way in patients, and even in mouse models their analysis is usually expensive, time consuming and technically challenging or sometimes even impossible. In contrast, their functional analysis in *D. discoideum* and other simple model organisms is often easier, faster and cheaper [21], [22]. Despite its lower complexity, *D. discoideum* is very similar to higher eukaryotes in many cellular aspects and for example is increasingly used to study autophagy and human disease genes [23], [24]. A major advantage of *Dictyostelium* is a large toolbox for the generation of mutants [25]. Previous work in *D. discoideum* showed that autophagy is required for normal development. Autophagy mutants were generated in six core autophagy genes and all mutants displayed developmental defects albeit of variable severity [26], [27]. ATG9 deficient cells had a pleiotropic phenotype and displayed severe defects in growth, phagocytosis and development [28].

Here we report on the analysis of *D. discoideum* strains that ectopically express p97 as well as p97^R155C^ fused to RFP in AX2 wild-type and ATG9^KO^ cells. The AX2/p97^R155C^-RFP strain mirrors the situation in heterozygous patients, while ATG9^KO^/p97^R155C^-RFP cells allow the investigation of mutant p97 in an autophagy deficient background. We provide genetic, biochemical, and cell biological evidence that p97 functionally links proteasomal activity and autophagy in *Dictyostelium*.

## Materials and Methods

### *Dictyostelium* Strains, Growth, Development, and Phototaxis

*D. discoideum* strain AX2 was used as wild-type strain. Generation of ATG9 knock-out cells has been described previously [28]. Strains expressing p97-RFP and p97^R155C^-RFP were generated by transformation of AX2 and ATG9^KO^ cells [28], respectively, with an expression construct encoding the fusion protein in the p389-2 mRFPmars vector [29]. Wild-type and mutant strains were grown at 21°C in liquid nutrient medium on plates (90 mm diameter) or with shaking at 160 rpm [30] or on SM agar plates with *Klebsiella aerogenes* [31]. The analysis of cell growth in shaking culture and on *K. aerogenes* as well as development and phototaxis experiments were carried out as described [28].

### Vector Construction and Transformation

The vectors for expression of full-length p97 and p97^R155C^ as RFP fusion proteins in *D. discoideum* were constructed using the p389-2 mRFPmars vector [29]. Expression was under the control of the actin-15 promoter and actin-8 terminator. To express wild-type p97 fused to RFP, full length *Dictyostelium* p97 (DDB_G0288065) was amplified by PCR, cloned into the p389-2-mRFPMars vector and the sequence verified. The R155C mutation was introduced by site directed mutagenesis with the QuikChange® Site-Directed Mutagenesis Kit (Agilent Technologies) according to the instruction by the manufacturer and confirmed by sequencing. In both fusion proteins a linker of nine amino acids with the sequence GGSGGSGGS separated the RFP moiety from p97. The plasmids were introduced into AX2 wild-type cells and the ATG9^KO^ mutant by electroporation [32]. Transformants were selected in the presence of 10 µg/ml G418 (Gibco, Germany) and cloned on *K. aerogenes* as described [28]. Transformants that expressed the fusion proteins were identified by visual inspection under a fluorescence microscope followed by immunological detection of the expressed protein in Western blots. Transformants were selected for further experiments that expressed approximately equal amounts of the p97-RFP or p97^R155C^-RFP fusion protein, respectively.

### Antibody Generation, SDS-PAGE, Western Blotting and Protein Quantitation

For the generation of specific polyclonal antibodies (pAbs) against *D. discoideum* p97 (DDB_G0288065), sequences encoding amino acids 23–73 (resulting pAb p97_8_6841) and 254–310 (resulting pAb: p97_9_6574) were amplified and cloned into the pGEX-6P-1 expression vector. Sequences encoding full-length *D. discoideum* ATG8 (DDB_G0286191) (resulting pAb: ATG8_6080) were amplified and cloned into a pGEX-4T expression vector. The fusion proteins were expressed in *Escherichia coli* XL1 Blue or DH5α, purified using glutathione-sepharose beads, released through cleavage with either PreScission or thrombin protease and used for the immunization of rabbits (BioGenes GmbH, Germany). SDS-PAGE and Western blotting were essentially performed as described [33], [34]. The proteins of 2×10^5^ cells were separated per lane for SDS gel electrophoresis of total cell lysates. The generated p97 and ATG8 pAbs were used for Western blotting at a 1∶10,000 dilution. GFP was detected with monoclonal antibody K3-184–2 [35], RFP with a polyclonal RFP antibody at a 1∶50,000 dilution (to be published elsewhere), proteasomal subunit 5 (SU5) with a monoclonal antibody at a 1∶100 dilution [36], and ubiquitin with the P4D1 monoclonal antibody at a 1∶1000 dilution (NEB, Germany). Secondary antibodies used were anti-mouse and anti-rabbit IgG conjugated with peroxidase (POD) (Sigma, Germany) followed by chemiluminescence detection. Images were recorded and analyzed using the Fluorchem SP imaging system (Alpha Innotech, USA). The amounts of ATG8 and SU5 were determined densitometrically using the Spot Denso tool of the AlphaEaseFC software (Alpha Innotech, USA). Background values were subtracted and the resulting intensities normalized based on actin (mAb Act-1-7) [37] or α-tubulin (mAb YL1/2) [38] staining. Mean values and standard deviations of four independent experiments were calculated.

### Purification of Recombinant p97 and Native Gel-electrophoresis

For expression of full-length p97 and p97^R155C^ as GST fusion proteins, the above p97 cDNAs were cloned into pGEX-6P-1 (GE Healthcare) and transformed into *E. coli* XL1 Blue. Subsequent protein purification and cleavage of GST were essentially done as described [39]. Samples of the affinity purified proteins were subjected to SDS-PAGE as well as BN–PAGE (blue native PAGE) according to [40].

### Determination of Proteasomal Activity

Proteasomal activity assays of the different *D. discoideum* strains were performed using the established protocol from skeletal muscle tissue with minor changes [41]. i), protein extraction and quantitation: Frozen cell pellets containing 1×10^6^ cells were lysed by thawing on ice, immediately re-suspended in 500 µl of PBS containing 5 mM EDTA (PBSE) and particulate material sedimented by centrifugation at 13,000×*g* for 10 min. The supernatants were subjected to protein quantitation employing the fluorescence-based ProStain Protein Quantitation Kit (Active Motif, Belgium) with bovine serum albumin (100, 50, 25, 12.5, 6.25, 3.125, and 1.56 µg) as standard. Protein extraction buffer PBSE was used as blank. The supernatants were diluted 1∶5, 1∶10, and 1∶20 and mixed with a fluorescent dye to a final volume of 200 µl in a non-transparent black 96-well plate (Nunc, Germany). The reactions were incubated at room temperature for 30 min and fluorescence was measured three times at 485 nm excitation and 590 nm emission wavelengths in an Infinite M1000 plate reader (Tecan, Switzerland). The coefficients of variation (r^2^) for the standard curves were between 0.97 and 0.99. ii), proteasome activity assay: For the proteasome activity assay, protein concentrations were adjusted to 0.2 mg/ml with PBSE. 50 µl (10 µg) of the protein lysate were added to 50 µl of the luminescent reagent containing the Ultra-Glo™ Luciferase and the signal peptide specific for chymotrypsin-like activity coupled to luciferin (Promega, Germany). To differentiate between unspecific background activity and proteasomal activity, the proteasomal inhibitor MG132 was added in control experiments at a final concentration of 100 µM. The reaction mixtures were mixed for 10 s and the luminescence signal was detected for two h in 10 min intervals in an Infinite M1000 plate reader (Tecan, Switzerland) using the luminescence setup and an integration time of 1 s. iii), calculation of the specific proteasomal activity: Protein lysates (3 µg) were separated by SDS-PAGE and proteins transferred onto nitrocellulose membranes by tank blotting over night at 4°C. Protein transfer was confirmed by Ponceau S staining. Membranes were blocked for 1 h at room temperature in TBS-T buffer (10 mM Tris/HCl pH 8.0, 150 mM NaCl, and 0.2% Tween 20) containing 5% milk powder and were probed with a monoclonal antibody directed against SU5 [36] followed by anti-mouse secondary antibody conjugated with peroxidase (Sigma, Germany) and chemiluminescence detection. The specific proteasomal activity was calculated after 60 or 120 min (depending on signal stability) by normalization with the amount of SU5 obtained from densitometric analysis as described above. Seven (AX2, ATG9^KO^, AX2/p97^R155C^-RFP, ATG9^KO^/p97^R155C^-RFP), four (AX2/p97-RFP), and three (ATG9^KO^/p97-RFP) independent experiments with duplicate samples were performed and mean values and standard errors calculated. The chymotrypsin-like activity of AX2 wild-type cells was set to 1.

### Fluorescence Microscopy

Immunofluorescence microscopy was done as described [28]. The following monoclonal and polyclonal antibodies, either undiluted or diluted in PTB (1× PBS, 0.1% Triton X-100, 0.1% BSA) buffer were used (dilution in brackets): monoclonal antibody, ubiquitin P4D1 (NEB, Germany) (1∶100); polyclonal antibodies, p97_8 (1∶100) and p97_9 (1∶100). Secondary antibodies were Alexa-fluor 488 goat anti-rabbit (1∶2,000) and Alexa-fluor 647 donkey anti-mouse (1∶2,000) (Invitrogen, Germany). The nuclei were stained with 4′,6-diamidino-2-phenylindole (DAPI, Sigma-Aldrich, Germany). Confocal images of fixed cells were recorded in sequential mode with a TCS SP5 laser scanning microscope (Leica, Germany) with a 100 × HCX PL APO NA 1.40 oil immersion objective. Excitation of Alexa-fluor 488 was at 488 nm, emission 500–550 nm; of Alexa-fluor 647 at 633 nm, emission 648–723 nm; and of DAPI at 405 nm, emission at 412–464 nm. Images were processed using the Leica Application Suite (LAS AF) software.

### Co-immunoprecipitation Experiments

*D. discoideum* cells to be used for co-immunoprecipitation experiments were grown at 21°C in 100 ml AX2 medium containing appropriate antibiotics. Log-phase cells (2–4×10^6^ cells/ml) were harvested by centrifugation (5 min, 500×*g*) and solubilized in 25 ml lysis buffer (30 mM 4-(2-hydroxyethyl)-1-piperazineethanesulphonic acid (HEPES) pH 7.5, 100 mM NaCl, 5 mM MgCl_2_, 2 mM ATP, 1 mM DTT, 0.5% Triton X-100, 1 mM PMSF and protease inhibitor cocktail (Roche, Germany)). The samples were homogenized by 20 strokes of a tightly fitting dounce homogenizer, cell debris was spun down for 20 min at 15,000×*g*, and supernatants were pre-cleared with 100 µl Protein A sepharose beads for 1 h at 4°C to remove protein that bound non-specifically to the beads. A polyclonal anti-RFP (7 µl) antibody was added to 100 µl Protein A sepharose beads for 2 h at 4°C. Afterwards, the beads were centrifuged (10 s, 500×*g*) and then blocked overnight with 5% BSA in 1× PBS on a rotating wheel. 7 ml pre-cleared cell lysate from different strains were incubated with the appropriate antibody bound beads for 90 min at 4°C. The samples were centrifuged and pellets were washed twice in lysis buffer and 5 times in washing buffer (lysis buffer without protease inhibitor cocktail). Finally, the samples were boiled for 5 min in 100 µl SDS-PAGE sample buffer, fractionated on a 10% SDS-polyacrylamide gel and either used for western blotting or stained with Coomassie brilliant blue.

## Results

### Generation of *Dictyostelium* Strains that Ectopically Express p97 Fused to RFP

**Figure 1:**
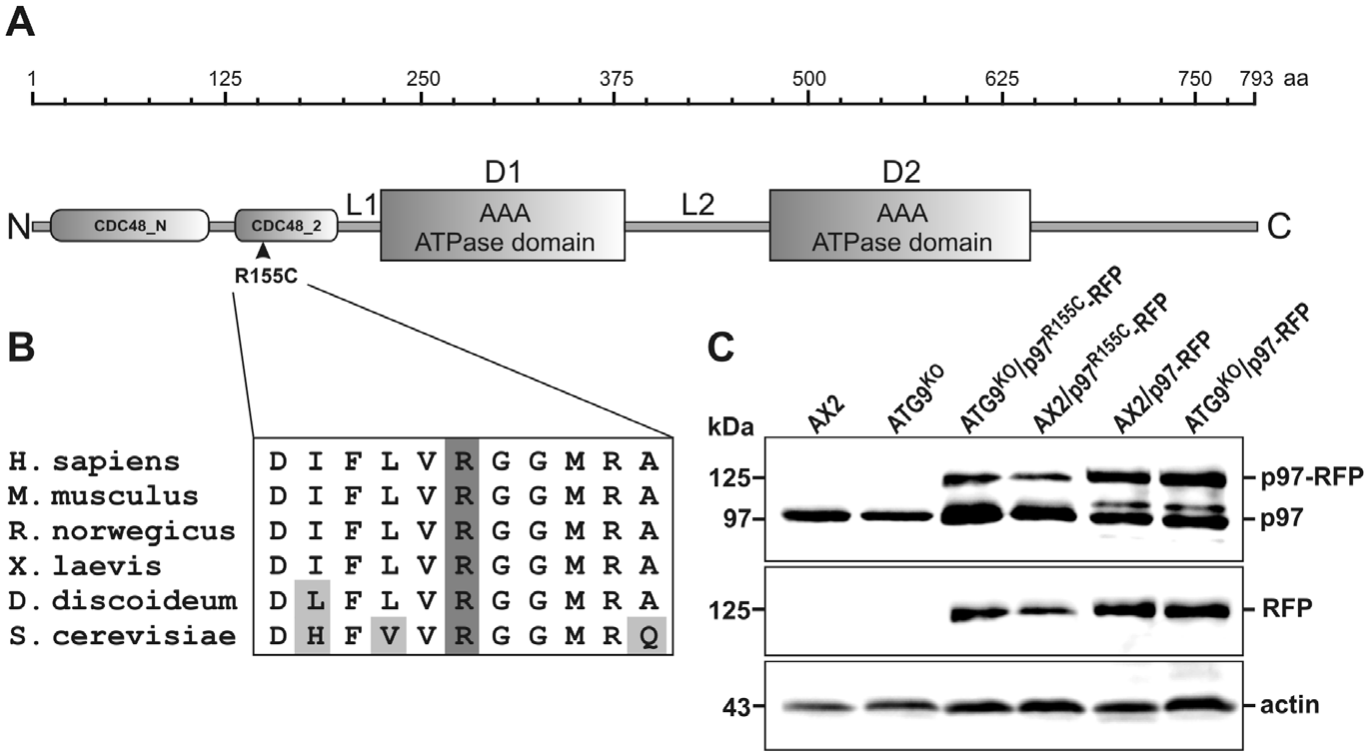
Domain structure of p97 and immuno-verification of mutant *Dictyostelium* strains. (**A**) Domain structure of p97. The 793 amino acid protein is composed of two N-terminal CDC48-like domains, followed by two AAA ATPase domains, D1 and D2, which are separated by the L2 linker region and a C-terminal region of approximately 160 amino acids. (**B**) The R155C mutation which causes IBMPFD in affected individuals is situated in the second CDC48-like domain. The region surrounding the arginine 155 is highly conserved from yeast to man. (**C**) Ectopic expression of p97-RFP and p97^R155C^-RFP in AX2 wild-type cells and in the ATG9^KO^ mutant. Top: Verification of expression of endogenous and RFP-fused p97 using pAb p97_8_6841. Middle: Verification of expression of RFP-fused p97 using a polyclonal RFP antibody. Bottom: loading control, actin.

p97, a member of the large AAA-ATPase family, has a tripartite structure comprising an N-terminal domain (CDC48) involved in ubiquitin binding and two central domains, D1 and D2, which bind and hydrolyze ATP (Fig. 1A). It is highly conserved from yeast to man and among vertebrates it is more than 95% identical. *Dictyostelium* p97 is 78% identical and 87% similar to the human ortholog, and yeast p97 still shares 68% sequence identity with the human and *Dictyostelium* counterparts (Table 1). To date, at least 20 unique p97 disease causing missense mutations have been reported. Most of these mutations are located in exons coding for the CDC48 domain with the R155C mutation being the most frequent [3], [5], [42]. The region surrounding the arginine is absolutely conserved in vertebrates and there is only one conservative I/L replacement in *D. discoideum* (Fig. 1B). In humans, the R155C mutation causes IBMPFD [3]–[5], ALS14 [6], or HSP [7], [8], however, the molecular consequences of the point mutation are hitherto unknown. Initially, we aimed to replace the single *Dictyostelium* p97 gene by the R155C mutant p97 variant fused to GFP via homologous recombination employing a knock-in strategy. Although we frequently obtained transformants expressing p97-GFP under the control of the endogenous p97 promoter, we were not able to isolate clones expressing p97^R155C^-GFP. We therefore changed our strategy and generated *Dictyostelium* strains, that ectopically express p97 or p97^R155C^ fused to RFP in either AX2 wild-type cells or the ATG9^KO^ mutant (Fig. 1C). Note, that the ectopic expression of p97^R155C^-RFP in haploid AX2 wild-type cells mimics the heterozygous situation of patients.

**Table 1:** Sequence identity and sequence similarity of p97 from different organisms.

	Hs	Mm	Rn	Xl	Sc	Dd
**Hs**		99/100	99/100	97/99	68/84	78/87
**Mm**			99/100	96/99	68/84	78/87
**Rn**				97/99	68/84	81/89
**Xl**					68/84	80/89
**Sc**						68/74

Sequence identity, left, and sequence similarity, right, was determined by aligning the corresponding protein sequences using BLAST align program (bl2seq) at the NCBI. Percentage values are given. Hs: *Homo sapiens*, *Mm: Mus musculus, Rn: Rattus norwegicus, Xl: Xenopus laevis, Sc: S. cerevisiae, Dd: D. discoideum.*

### Wild-type and Mutant p97 form Heteromeric Complexes

**Figure 2:**
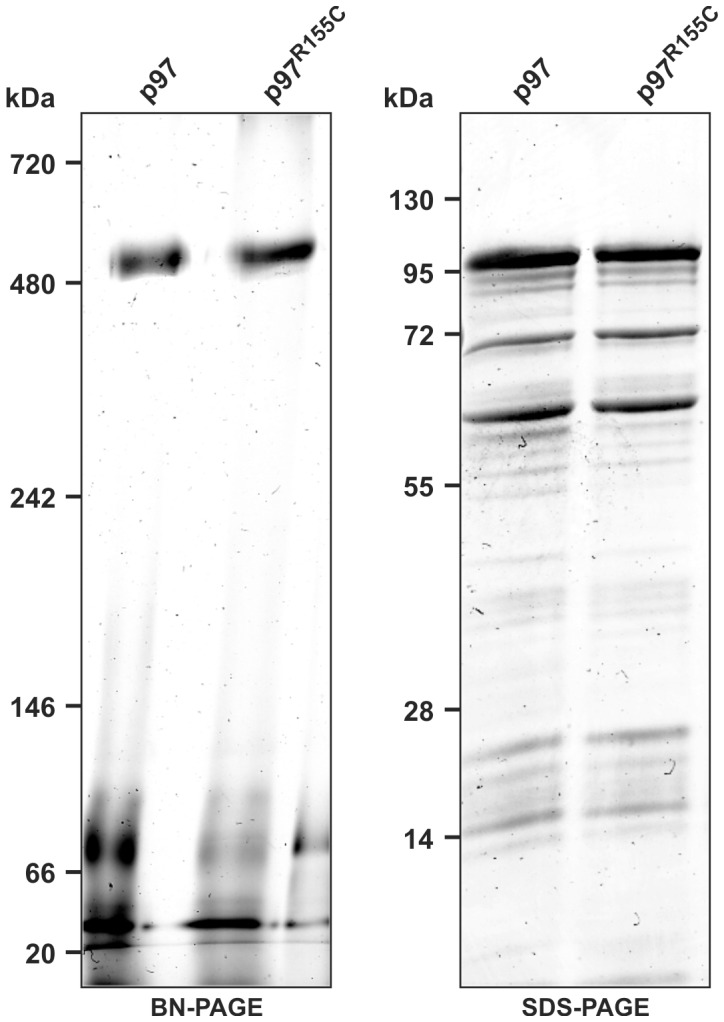
Purified recombinant p97 and p97^R155C^ form hexamers. Purified recombinant p97 and p97^R155C^ were subjected to either blue-native (**left image**) or denaturing (**right image**) gel electrophoresis followed by Coomassie brilliant blue staining. Under native conditions, wild-type and mutant p97 migrate at a position corresponding to approximately 600 kDa.

Human p97 has been shown to assemble into functional hexamers *in vivo* [9]. We expressed and purified *Dictyostelium* wild-type as well as R155C mutant p97 from bacteria and subjected them to native and denaturing gel electrophoresis. The latter showed that both proteins migrated at approximately 100 kDa corresponding to the molecular mass of the monomer. Under native conditions, both wild-type p97 and the R155C mutant migrated at approximately 600 kDa which is in good agreement with the formation of hexamers (Fig. 2).

**Figure 3:**
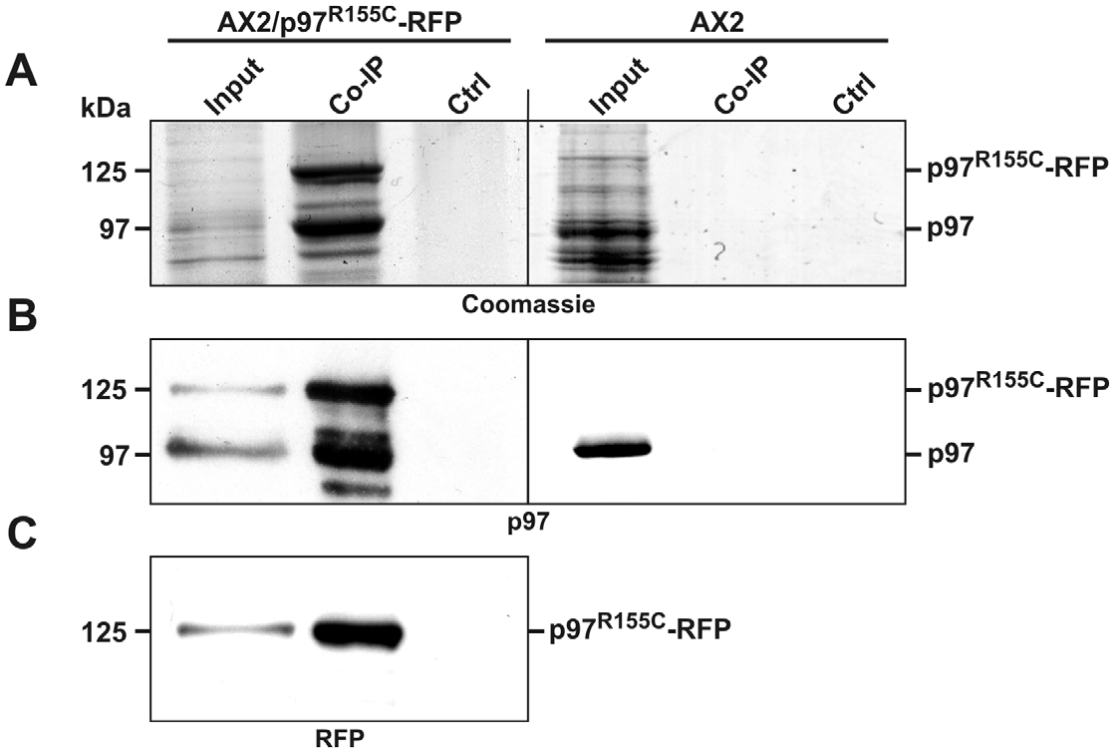
p97 and p97^R155C^ form heteromers in vivo. (**A**) Coomassie stained SDS-PAGE gel of a p97 co-immunoprecipitation experiment. Input: soluble cell lysate of either AX2/p97^R155C^-RFP (**left column**) or AX2 (**right column**) cells; Control (ctrl): beads incubated with bovine serum albumin instead of the polyclonal RFP antibody; CoIP: co-precipitated proteins by the RFP antibody. (**B**) Immunoblot verification of the presence of p97 and p97^R155C^-RFP in the immunoprecipitate and input. (**C**) Immunoblot verification of the presence of p97^R155C^-RFP.

Next, we investigated whether p97^R155C^-RFP associates with endogenous p97. Co-immunoprecipitation experiments using cell lysates and polyclonal antibodies against RFP clearly showed that p97 and p97^R155C^-RFP bind to each other (Fig. 3). The ratio of the two proteins in the immune precipitate was about the same as in total cell lysates (compare Fig. 1C and 3B), indicating that neither the R155C mutation nor the RFP tag cause problems in the formation of hexamers. Thus, the majority of the p97 complexes are expected to be heteromers of both protein variants.

### Mutant Strains Display Growth Defects

**Figure 4:**
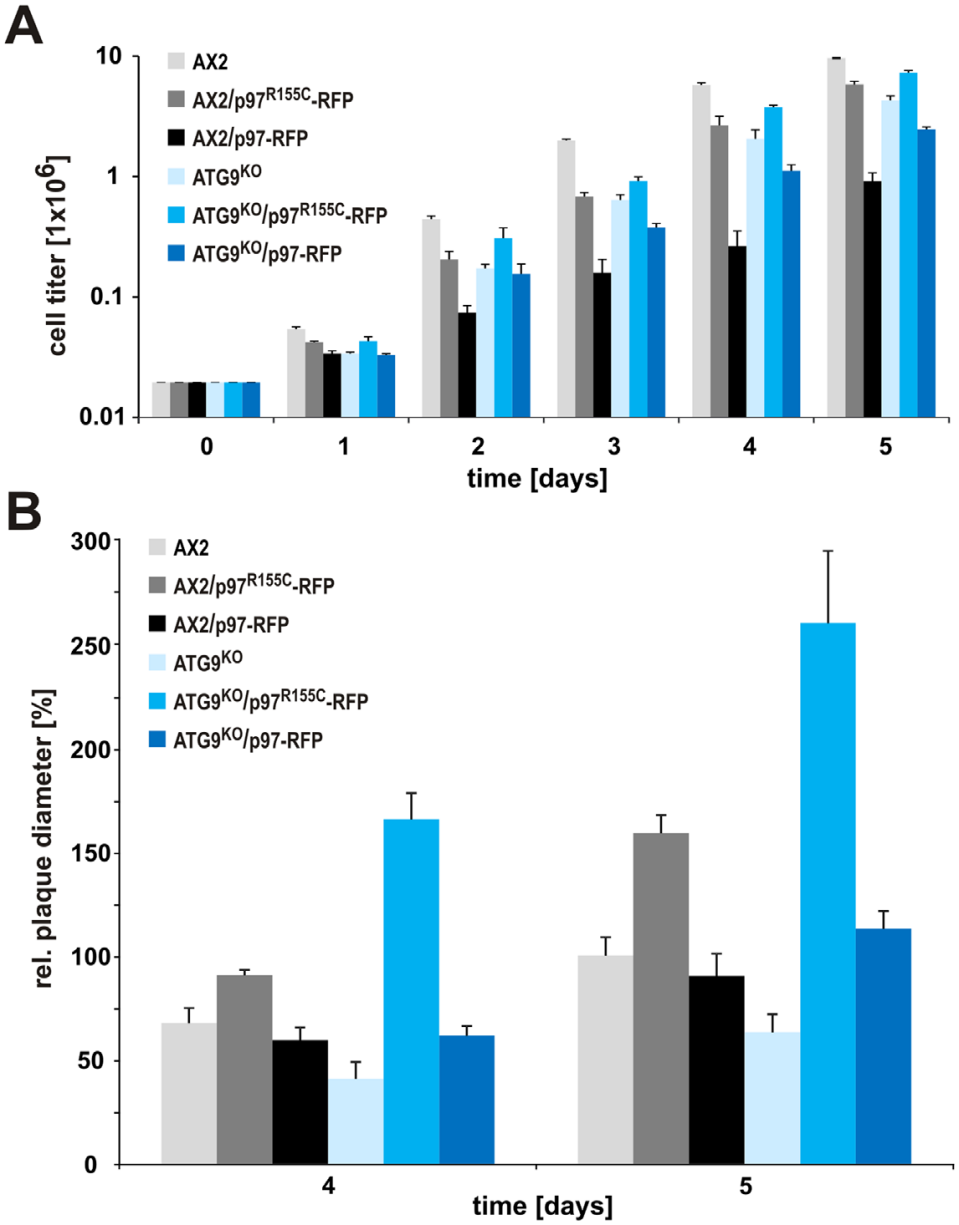
Cell growth in shaking culture and on *Klebsiella aerogenes* are altered in mutant strains expressing p97-RFP or p97^R155C^-RFP. (A) Strains expressing p97-RFP or p97^R155C^-RFP display specific growth defects in shaking culture. Please note the logarithmic scale of the y-axis. (B) Growth on *K. aerogenes* lawns. Mutation specific and dose dependent effects are seen in both wild-type and ATG9^KO^ strains. Growth of AX2 on day 5 was set to 100%.

In the following, the cellular consequences of heteromeric p97 complexes were investigated with a number of assays in wild-type and autophagy 9 minus cells. AX2 wild-type cells can be grown in shaking culture and also on bacterial lawns of e.g. *Klebsiella aerogenes*. When cell growth was measured over a period of five days in liquid medium, we observed a strong growth defect in the ATG9^KO^ compared to AX2 wild-type cells, consistent with earlier results [28]. Expression of p97^R155C^-RFP in the ATG9^KO^ background caused a partial rescue of this phenotype, while expression of p97-RFP resulted in an even more pronounced growth defect. Expression of p97^R155C^-RFP in the background of AX2 wild-type cells resulted in a similar growth defect as observed in the ATG9^KO^ strain. Upon expression of p97-RFP this defect was even stronger (Fig. 4A; note the logarithmic scale).

Next we analyzed growth on a lawn of *K. aerogenes* by determining the plaque diameter of single clones after 4 and 5 days of growth. We measured a significantly smaller plaque size for ATG9^KO^ as compared to AX2 wild-type cells, corresponding to a phagocytosis defect reported in a previous study [28]. In contrast, expression of p97^R155C^-RFP in the ATG9^KO^ background resulted in considerably larger plaques, whereas p97-RFP only led to a slight increase compared to AX2 wild-type cells. A smaller, however, highly significant increase in plaque size was also seen in AX2 cells expressing p97^R155C^-RFP while the expression of wild-type p97-RFP in AX2 wild-type cells had no significant influence (Fig. 4B).

### p97 is Present in Ubiquitin-positive Protein Aggregates in the ATG9^KO^ but not the ATG9^KO^/p97^R155C^-RFP Strain

**Figure 5:**
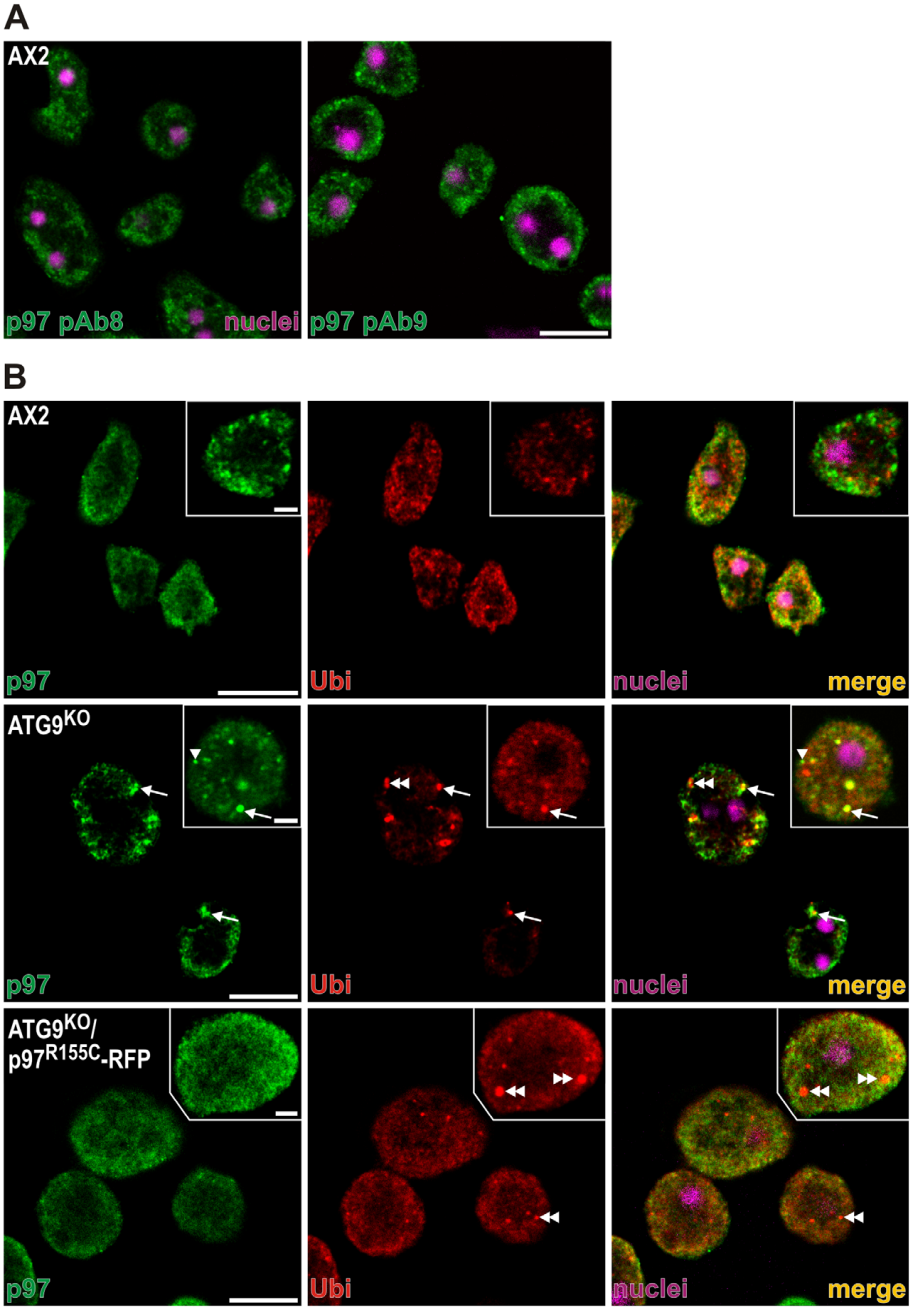
Subcellular localization of p97 and its co-localization with ubiquitin in wild-type and mutant strains. (**A**) Visualization of subcellular localization of p97 in AX2 wild-type cells with polyclonal antibodies p97_8_6841 directed against amino acids 23–73 and p97_9_6574 directed against amino acids 254–310. (**B**) Subcellular localization of p97 (**left panel**) using the p97_8_6841 polyclonal antibody and ubiquitin (**middle panel**) using the P4D1 monoclonal antibody (NEB, Germany) in AX2 wild-type cells and mutant strains. Merged images and DAPI staining to visualize nuclei (**right panel**). **Upper row**: AX2 wild-type cells; **middle row**: ATG9^KO^ mutant; **bottom row**: ATG9^KO^/p97^R155C^-RFP double mutant. Please note that ubiquitin positive protein aggregates frequently co-localize with p97 in the ATG9^KO^ mutant (arrows) but not in the ATG9^KO^/p97^R155C^-RFP double mutant (double arrowheads). The ATG9^KO^ mutant also contains protein aggregates that are either positive for p97 (arrowhead) or ubiquitin (double arrowhead). Cells were fixed with cold methanol and stained with the indicated antibodies. Scale bars are 10 µm and 2 µm in inset.

In AX2 wild-type cells, immunofluorescence studies employing two polyclonal antibodies that are directed against different regions of the p97 protein revealed a punctate cytoplasmic staining pattern (Fig. 5A). Since p97 is a crucial component in the delivery of poly-ubiquitinylated proteins to the proteasome, we investigated its co-localization with ubiquitin. In AX2 cells we rarely detected an overlap between p97 and ubiquitin. Both proteins were localized throughout the cytoplasm (Fig. 5B, top row). In ATG9^KO^ cells we frequently observed large protein aggregates that often stained with p97 polyclonal and ubiquitin monoclonal antibodies (arrow), however, some aggregates were only positive for p97 (arrowhead) or ubiquitin (double arrowhead), indicating dynamic recruitment of these proteins to the aggregates (Fig. 5B, middle row). ATG9^KO^ cells that expressed p97^R155C^-RFP displayed ubiquitin-positive protein aggregates in a similar frequency, but we no longer detected any co-localization with p97 (Fig. 5B, bottom row, double arrowhead).

### Ectopic Expression of p97 and p97^R155C^ Influence Protein Ubiquitinylation and Proteasomal Activity

**Figure 6:**
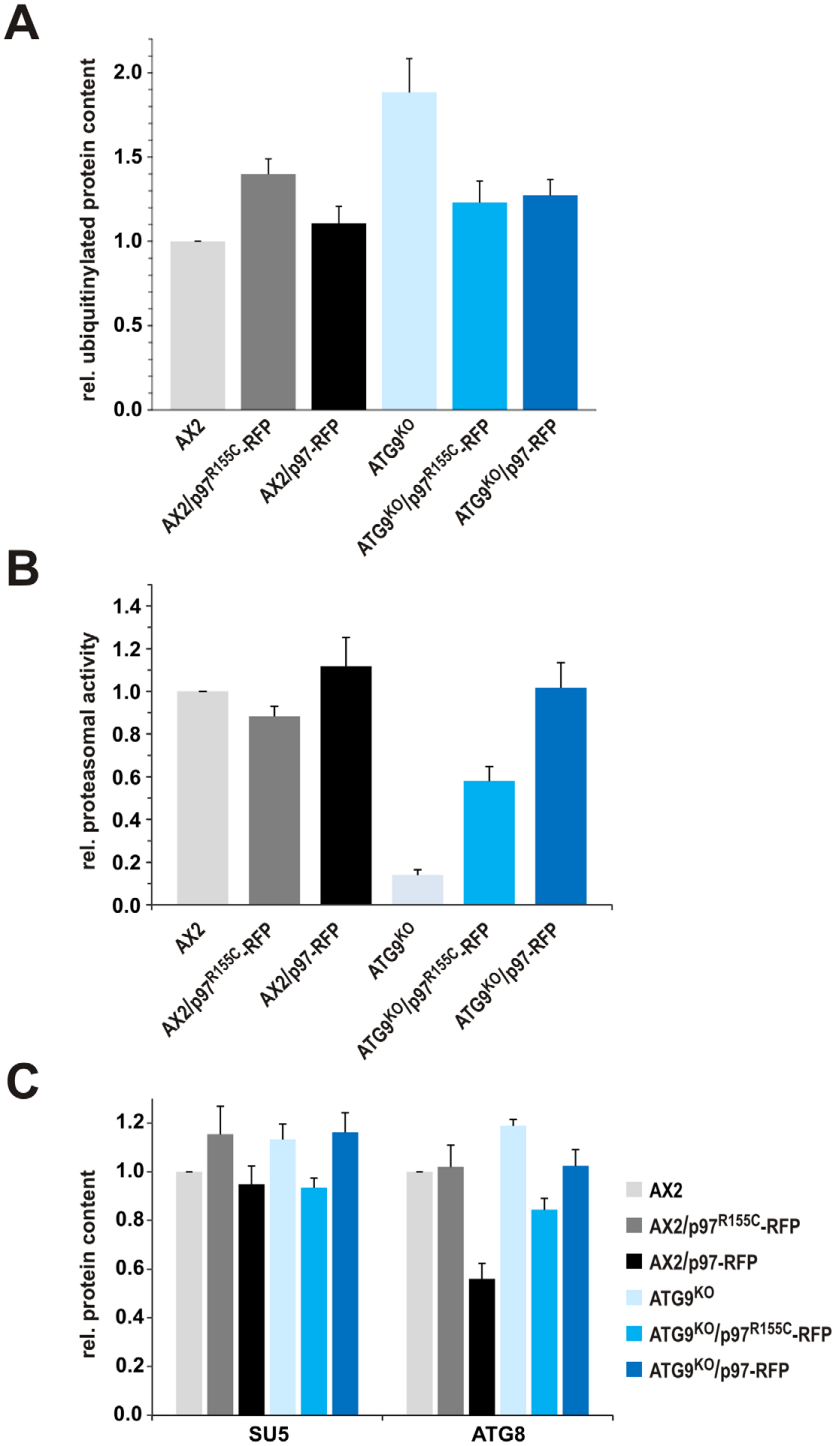
Ubiquitinylation, proteasomal activity and levels of SU5 and ATG8. Strains expressing p97-RFP or p97^R155C^-RFP display specific changes in ubiquitinylation (**A**), proteasomal activity (**B**), and levels of SU5 and ATG8 (**C**). Values of AX2 have been set to 1; normalization was to actin (see Fig. S1). For detection of ubiquitin, SU5, and ATG8 in Western blots the monoclonal antibodies P4D1 (NEB, Germany) and proteasomal subunit 5 (SU5) [36] as well as the ATG8_6080 polyclonal antibody were used, respectively. The proteasomal activity assay was performed by adding the Ultra-Glo™ Luciferase and the signal peptide specific for chymotrypsin-like activity coupled to luciferin (Promega, Germany) to cell lysates. Proteasomal activity was normalized to proteasome content, and the chymotrypsin-like activity of AX2 was set to 1.

The presence of ubiquitin- and p97-positive protein aggregates in ATG9^KO^ cells prompted us to investigate overall levels of ubiquitinylated proteins in the different strains. Ubiquitin is a highly conserved protein that is covalently linked to many cellular proteins to mark them for degradation by the 26S proteasome. We used the mouse monoclonal antibody P4D1 which recognizes ubiquitin, poly-ubiquitin and ubiquitinylated proteins and cross-reacts with *Dictyostelium* ubiquitin to detect ubiquitinylated proteins in whole cell lysates. In AX2 lysates we detected a large number of ubiquitinylated proteins ranging in size from approximately 400 to 15 kDa. The pattern of ubiquitinylated proteins was similar in lysates of the mutant strains. However, we observed a moderate increase in AX2/p97^R155C^-RFP lysates and a strong signal increase in ATG9^KO^ lysates. Expression of either p97 or p97^R155C^ RFP fusion proteins in the ATG9^KO^ background resulted in ubiquitinylation levels similar to AX2 wild-type cells (Fig. 6A; Fig. S1A).

Increased levels of ubiquitinylated proteins may lead to an induction of either or both of the two main protein degradation pathways, autophagy and the proteasomal system. We therefore measured the specific proteasomal activity, which was normalized to the proteasomal content. We observed a minor decrease of proteasomal activity in AX2/p97^R155C^-RFP and a minor increase in AX2/p97-RFP lysates. In ATG9^KO^ lysates we observed a nearly complete loss of the specific proteasomal activity, which could be partially or completely rescued by expression of p97^R155C^-RFP or p97-RFP, respectively (Fig. 6B).

In addition, we quantitated levels of the core autophagy protein ATG8(LC3) and of the proteasomal subunit 5 (SU5) in whole cell lysates of AX2 and the mutant strains. In the setting of the markedly increased levels of ubiquitinylated proteins and the loss of proteasomal activity in ATG9^KO^ cells, we detected a moderately increased amount of ATG8. Presence of p97^R155C^-RFP in ATG9^KO^ cells however moderately decreased ATG8 levels as compared to AX2 cells. A further consistent finding was the strong decrease of ATG8 levels in AX2/p97-RFP cells. Expression of p97^R155C^-RFP caused a moderate increase of the SU5 protein level in AX2 wild-type cells. The level of SU5 was also moderately increased in the ATG9^KO^ strain. Here, expression of p97^R155C^-RFP but not p97-RFP led to reduction of SU5 to wild-type levels (Fig. 6C; Fig. S1B).

### Expression of p97^R155C^-RFP Causes a Phototaxis Defect in AX2 Cells and Rescues the Lack of Phototaxis in the ATG9^KO^ Strain

**Figure 7:**
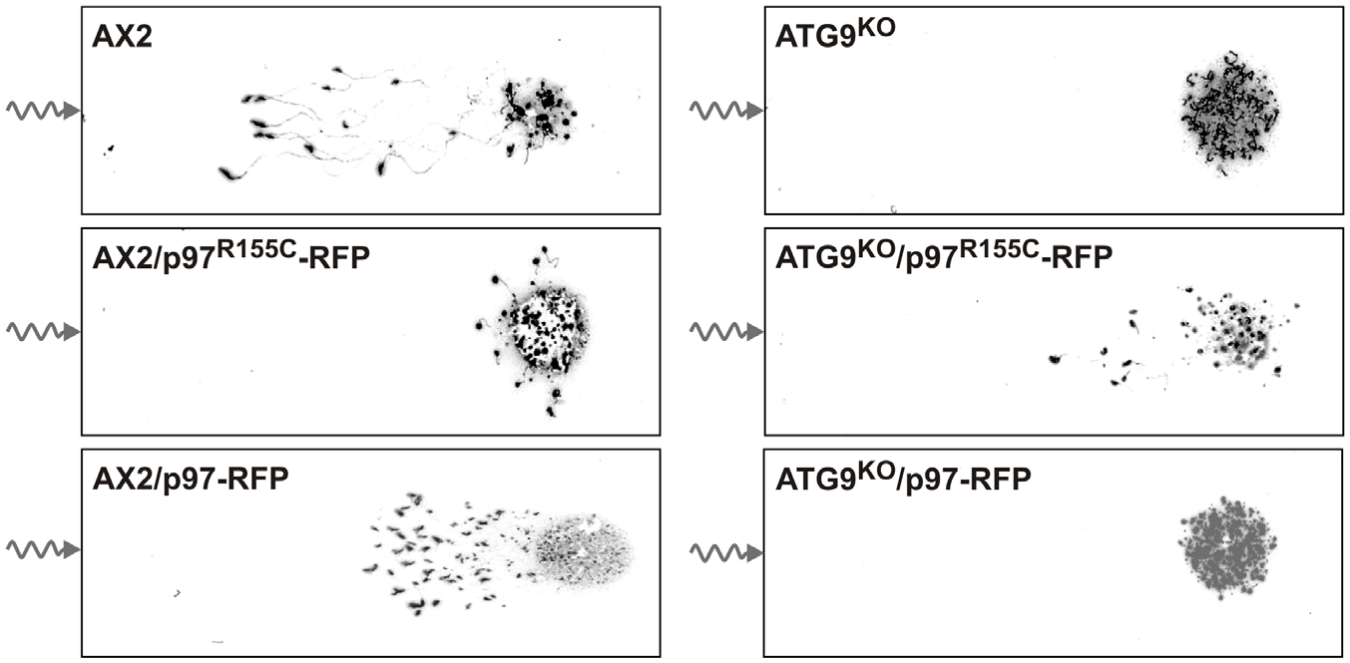
Expression of p97^R155C^-RFP impairs phototaxis in AX2 cells and rescues the phototaxis defect of ATG9^KO^ cells. The ability of AX2 wild-type and of mutant slugs to migrate towards a light source (wavy line) was tested. While AX2 slugs (**top left image**) and AX2 slugs expressing p97-RFP (**bottom left image**) nicely migrated towards the light source, phototactic ability was severely impaired in the AX2/p97^R115C^-RFP strain (**middle left image**). In the ATG9^KO^ strain phototaxis is completely lost (**top right image**). While expression of p97^R155C^-RFP in the ATG9^KO^ strain partially rescued the phototactic ability (**middle right image**), no rescue of phototactic ability was observed upon expression of p97-RFP (**bottom right image**). The phototaxis assay was performed as described [28].

Previously, we had found that ATG9^KO^ slugs had completely lost the ability to migrate towards light while AX2 slugs nicely phototax (Fig. 7, top images and [28]). When the AX2/p97^R155C^-RFP mutant was assayed for phototactic behavior, a strong defect was seen as slugs moved only short distances and also directionality was severely limited. In contrast, expression of p97^R155C^-RFP in the ATG9^KO^ strain partially rescued the phototactic ability (Fig. 7, middle images). As controls, we tested slugs of AX2 as well as the ATG9^KO^ strains expressing wild-type p97 fused to RFP. The former slugs migrated nearly as well as the AX2 wild-type, while the latter were indistinguishable from the ATG9^KO^ (Fig. 7, bottom images).

### Expression of p97^R155C^-RFP in ATG9 Deficient Cells Completely Rescues Fruiting Body Formation

**Figure 8:**
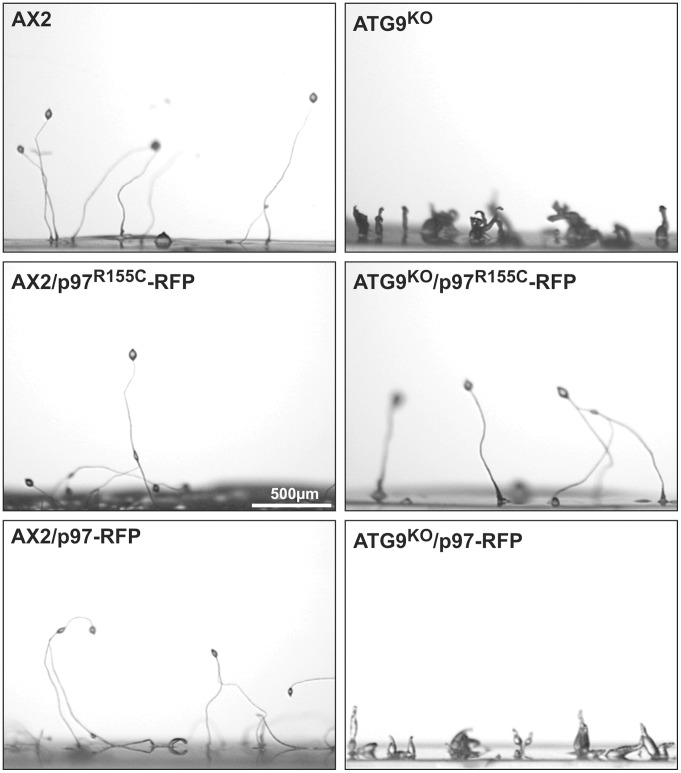
Expression of p97^R155C^-RFP rescues fruiting body formation in ATG9^KO^ cells. Neither expression of p97-RFP nor p97^R115C^-RFP changed fruiting body formation in AX2 wild-type cells (**left column**). In the ATG9^KO^ strain fruiting body formation is completely lost (**top right image**). While expression of p97^R155C^-RFP in the ATG9^KO^ strain fully rescued the fruiting body formation (**middle right image**), no obvious rescue of fruiting body formation was observed upon expression of p97-RFP (**bottom right image**). The assay was performed as described [28].

Ectopic expression of wild-type p97-RFP and mutant p97^R155C^-RFP had no effect on fruiting body formation in the AX2 wild-type background (Fig. 8, left column). ATG9^KO^ cells displayed a severe developmental defect and only could generate extremely small and misshaped fruiting bodies (Fig. 8, top right image and [28]). Expression of p97^R155C^-RFP in this strain completely rescued this phenotype and led to the formation of normal fruiting bodies (Fig. 8, right middle image). In contrast, the ATG9^KO^/p97-RFP control strain had a similar phenotype as the parent ATG9^KO^ strain (Fig. 8, right bottom image).

## Discussion

The goal of this study was to analyze cellular consequences of the expression of the disease causing p97^R155C^ point mutation in the model organism *Dictyostelium*. In a first approach, we aimed to replace the single *Dictyostelium* p97 gene by the p97^R155C^ variant fused to GFP by means of a knock-in strategy. More than 100 GFP-positive clones were tested for expression of the point mutation, however all expressed wild-type p97 fused to GFP. The lack of clones expressing p97^R155C^-GFP points towards a dramatic disadvantage or lethality of such a strain. It underscores the essential function of the R155 residue and mirrors the reported lethality of its disruption in yeast and the early embryonic lethality in VCP/p97 knock-out mice [43], [44]. We then generated strains ectopically expressing wild-type or mutant p97 in the AX2 wild-type (AX2/p97-RFP, AX2/p97^R155C^-RFP) and the ATG9^KO^ background (ATG9^KO^/p97-RFP, ATG9^KO^/p97^R155C^-RFP). Note, that *Dictyostelium* is a haploid organism and that expression of mutant p97 in addition to the endogenous p97 mirrors the situation in heterozygous IBMPFD patients. It was previously shown that the evolutionarily highly conserved human wild-type p97 [9] and p97 carrying the R155H mutation both assemble into hexamers [45]. To address this issue for *Dictyostelium* p97, we employed blue native PAGE and found that purified recombinant p97 as well as p97^R155C^ formed hexamers. Next, we performed co-immunoprecipitation experiments in strains ectopically expressing p97^R155C^ which clearly demonstrated that wild-type and mutant p97 assemble into heteromers *in vivo*. Both findings strongly suggest the presence of hexamers with variable ratios of wild-type and mutant p97 in p97/p97^R155C^ expressing strains.

Western blotting of lysates from AX2/p97^R155C^-RFP showed an increase of poly-ubiquitinylated proteins, which mirrors the finding in IBMPFD muscle tissue [46] and is in agreement with a role of wild-type p97 in ubiquitin-proteasome mediated protein degradation [12]. Moreover, our immunofluorescence analyses revealed protein aggregates in ATG9^KO^ cells that stained with antibodies against ubiquitin and p97, while protein aggregates in the ATG9^KO^/p97^R155C^-RFP strain were not immunoreactive for p97. This indicates that p97/p97^R155C^ hexamers are no longer able to associate with protein aggregates in *Dictyostelium*, probably because of problems to bind one or more specific adaptor proteins. In agreement with such a hypothesis a recent report suggested imbalanced co-factor binding to p97 as a key pathological feature of IBMPFD [47]. Furthermore, we found a severe defect of proteasomal activity in the ATG9^KO^ strain that was partially or completely rescued upon expression of p97^R155C^ or p97, respectively. A knock-down of p97 in HeLa cells was reported to lead to the accumulation of poly-ubiquitinylated proteins [48] and a reversible inhibitor of p97 impaired proteasomal and autophagy protein degradation pathways [49].

**Figure 9:**
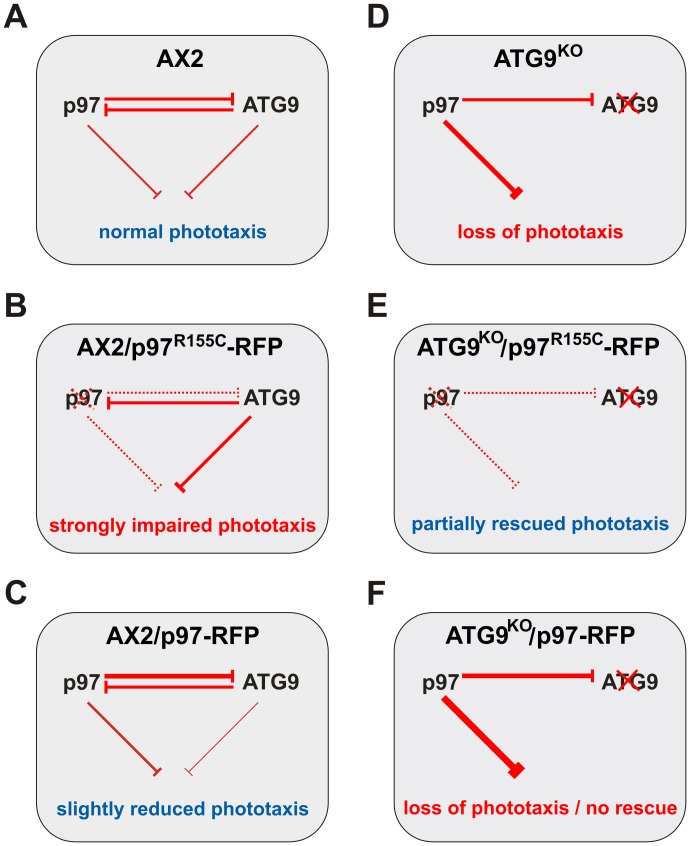
Interaction and mutual inhibition of p97 and ATG9. (**A**) The analysis of phototactic ability illustrates mutual inhibition of p97 and ATG9 in conjunction with inhibition of this process in AX2 cells. (**B**) Expression of p97^R155C^ in AX2 wild-type cells frees ATG9 to exert its inhibitory activity. (**C**) Expression of wild-type p97 in wild-type AX2 cells results in slightly reduced phototaxis which can be explained by a dose dependent effect. (**D**) In the absence of ATG9, its inhibitory activity versus p97 is lost leaving p97 free to strongly inhibit phototaxis. (**E**) Partial rescue of phototaxis in the ATG9^KO^/p97^R155C^-RFP double mutant. (**F**) Since phototaxis is already lost in ATG9^KO^ cells, the expression of p97-RFP cannot lead to a further impairment. Line width correlates with the strength of the inhibitory effect. Dotted line, residual activity. Please refer to the Discussion section for further details on this model of interaction and mutual inhibition.

The importance of the amino acid R155 for the cellular function of p97 became evident from the analyses of the two strains that ectopically expressed p97^R155C^. As controls, we included the two strains expressing wild-type p97 fused to RFP, i.e. AX2/p97-RFP and ATG9^KO^/p97-RFP. This setting allowed distinguishing between mutation and dose dependent cellular effects. Except for the proteasomal activity, where the p97 effect was more pronounced as compared to p97^R155C^, we always observed strong mutation specific effects. However, all cellular processes we have analyzed also showed a certain dependency on the p97 expression level (Table 2). Based on the data from our study, we propose i) that p97 and ATG9 directly or indirectly interact and ii) that they mutually inhibit each other (Fig. 9). Our proposed model is best illustrated by the phototaxis phenotypes. In the absence of ATG9, its inhibitory activity versus p97 is lost leaving p97 free to strongly inhibit phototaxis (Fig. 7, top right image; Fig. 9D). The partial rescue of phototaxis in the ATG9^KO^/p97^R155C^-RFP double mutant (Fig. 7, middle right image; Fig. 9E) suggests that the point mutation renders p97 inactive. The result can be explained if we assume that either p97/p97^R155C^-RFP hexamers have severely restricted functionality or are no longer functional at all. However, the latter case would require a fraction of functional p97 hexamers not containing the p97^R155C^ mutant. Consistent with this interpretation, expression of wild-type p97-RFP in the ATG9^KO^ background did not rescue phototaxis (Fig. 7, bottom right image; Fig. 9F). According to our proposed model, the expression of p97^R155C^-RFP in the AX2 wild-type situation led to strongly impaired phototaxis (Fig. 7, middle left image; Fig. 9B). The observed slight reduction of phototaxis upon expression of wild-type p97-RFP in addition to the endogenous p97 in AX2 wild-type cells is consistent with a dose dependent effect and further supports our model of mutual inhibition (Fig. 7, bottom left image; Fig. 9C).

**Table 2:** Simplified overview of p97 mutation and dose dependent effects.

analysis of cellular process		effects of p97	
		Mutationspecific	Dosedependent
cell growth	shaking culture	✓	✓
	*Klebsiella* lawns	✓	✓
colocalization with ubiquitin		✓	
ubiquitinylation of proteins		✓	✓
proteasomal activity		✓	✓
SU5 protein level		✓	–
ATG8 (LC3) protein level		✓	✓
phototaxis behaviour		✓	✓
fruiting body formation		✓	–

We used our proposed model of the p97– ATG9 mutual inhibition to predict the expected effects of the expression of p97 and p97^R155C^ in AX2 wild-type and ATG9^KO^ cells and compared them with our further experimental findings (Table 3). In AX2 derived strains, the experimental findings well agree with the predicted effects, however, with an inverse effect in three investigated cellular processes. In ATG9^KO^ derived strains, the patterns are more complex. The model seems to be valid for the expression of p97^R155C^, which always leads to a partial or full rescue of the ATG9^KO^ phenotype. In case of the ectopic expression of wild-type p97-RFP in the ATG9^KO^ background, the model correctly predicts the findings in half of the experiments. Note, however, that in two of the four results which do not agree with the model, the expression of p97-RFP and p97^R155C^-RFP still induce the expected alternating effects (Table 3, label “no^1)^”). This indicates that the *in vivo* situation is more complex. In our model of mutual inhibition, the individual effects of p97 and ATG9 for a downstream cellular process are taken as inhibitory. This assumption is consistent with most experimental results. However, p97 and/or ATG9 could also have a downstream stimulatory effect or even no effect on a specific cellular process.

**Table 3:** Predicted and observed experimental outcomes of the expression of p97 and p97^R155C^ in AX2 wild-type and ATG9^KO^ cells.

analysis of cellular process		AX2 derived strains					ATG9^KO^ derived strains				
		wild-type	p97^R155C^-RFP		p97-RFP		ATG9^KO^	p97^R155C^-RFP		p97-RFP	
			exp result	model validity	exp result	model validity		exp result	model validity	exp result	model validity
cell growth	shaking culture	ref value	↓	ok	↓↓	ok	ref value	↑	ok (rescue)	↓	ok
	*Klebsiella* lawns	ref value	↑	inverse	→	ok	ref value	↑↑	ok (rescue)	↑	*no^1)^*
ubiquitinylation of proteins		ref value	↑↑	inverse	→	ok	ref value	↓↓	inverse (rescue)	↓↓	*no*
proteasomal activity		ref value	↓	ok	→	ok	ref value	↑	ok (rescue)	↑↑	*no*
SU5 protein level		ref value	↑	inverse	→	ok	ref value	↓	inverse (rescue)	→	*ok*
ATG8 (LC3) protein level		ref value	→	ok	↓↓	ok	ref value	↓↓	inverse (rescue)	↓	*no^1)^*
phototaxis behaviour		ref value	↓↓	ok	↓	ok	ref value	↑↑	ok (rescue)	→	ok
fruiting body formation		ref value	→	ok	→	ok	ref value	↑↑	ok (rescue)	→	ok
**predicted changes based on model**		ref value		→/↓		→/↓	ref value		↑ (rescue)		→/↓

The table summarizes the predicted changes based on the model of p97 and ATG9 interaction and mutual inhibition as illustrated in Fig. 9. Experimental results in black; model validity in green; “no” and “no” with uppercase “1)”, see Discussion section; “ref value”, changes were separately compared to wild-type and ATG9^KO^ backgrounds; ↑, ↓, →, increase, decrease or no change with respect to the reference value. Inverse, inverted mutation specific effect. Rescue, partial or full rescue of the ATG9^KO^ phenotype.

Here, we provide genetic, biochemical and cell biological evidence that p97 and autophagy via ATG9 are functionally linked in *Dictyostelium*. The interaction and mutual inhibition of p97 and the core autophagy protein ATG9 is the key that sets the course for the proteasomal or autophagy pathway. Moreover, there is a delicate balance between the two major protein degradation pathways, proteasomal degradation and autophagy. In *Dictyostelium*, our model opens the possibility to search for proteins that interact with p97 in a R155 dependent manner in order to generate a more coherent picture of the complex pathology of p97 diseases. With respect to future treatment concepts for human p97 diseases, not only an induction of proteasomal activity or autophagy flux but also their inhibition in a certain cellular process might result in an attenuation of the disease phenotype.

## Supporting Information

Figure S1 **Levels of ubiquitinylated proteins, SU5, and ATG8(LC3).** For detection and quantitation of ubiquitin, SU5, ATG8, and actin in Western blots the monoclonal antibodies P4D1 (NEB, Germany), proteasomal subunit 5 (SU5) [36], and Act-1-7 [37] as well as the ATG8_6080 polyclonal antibody were used, respectively.
(TIF)
