# Correction: Heteromeric p97/p97^R155C^ Complexes Induce Dominant Negative Changes in Wild-Type and Autophagy 9-Deficient *Dictyostelium* strains

**DOI:** 10.1371/journal.pone.0199548

**Published:** 2018-06-18

**Authors:** Khalid Arhzaouy, Karl-Heinz Strucksberg, Sze Man Tung, Karthikeyan Tangavelou, Maria Stumpf, Jan Faix, Rolf Schröder, Christoph S. Clemen, Ludwig Eichinger

In the original article published in 2012, it was stated that stable ectopic expression of the p97^R155C^ mutant in *Dictyostelium discoideum* AX2 wild-type as well as autophagy 9-deficient (ATG9^KO^) cells caused the observed phenotypes [1]. Further work revealed that the respective *D*. *discoideum* p97-RFP expression plasmid used for transformation was confused by mistake. Unfortunately, it encoded an additional p97 point mutation changing the 221 amino acid residue from glutamate to lysine. The authors regret the mistake, and as a consequence, the title of the manuscript must be corrected to “Heteromeric p97/p97^R155C/E221K^ complexes induce dominant negative changes in wild-type and autophagy 9-deficient *Dictyostelium* strains”. Furthermore, the term “p97^R155C^” has to be replaced with “p97^R155C/E221K^” in the text and all figures, as the reported data were due to *D*. *discoideum* p97 containing these two point mutations. Please note that in the study by Arhzaouy *et al*. [1] the human p97 amino acid sequence numbering was chosen for the naming of the point-mutated *D*. *discoideum* p97 protein variant. The rationale behind this decision was to point out that the R154C point mutation in *D*. *discoideum* p97 corresponds to the R155C p97 missense mutation in human, which causes neurodegenerative diseases.

To clarify the influence of the additional E221K p97 mutation, we included this mutation in another, independent study in which we analyzed and compared wild-type p97 and the three point mutants, p97^R154C^, p97^E219K^ and p97^R154C/E219K^, on the biochemical level and only detected differences in p97 ATPase activity measurements [2]. In this study, we used the *D*. *discoideum* p97 amino acid sequence numbering, i.e. positions R155 and E221 of human p97 correspond to R154 and E219 of *D*. *discoideum* p97, respectively.

To furthermore re-evaluate whether the phenotypic changes observed in the ATG9^KO^ *D*. *discoideum* strain as reported in Arhzaouy *et al*. [1] were due to the expression of *D*. *discoideum* p97^R154C^, p97^E219K^ or p97^R154C/E219K^ protein variants (*D*. *discoideum* p97 amino acid sequence numbering), we newly generated two additional ATG9^KO^ strains that expressed the two single p97 mutants fused to RFP. Vector construction and transformation were done as described [1] and all p97 expression constructs used have been verified by sequencing. We repeated the analysis of fruiting body formation in all strains, i.e. AX2, ATG9^KO^, ATG9^KO^/p97^WT^-RFP, ATG9^KO^/p97^R154C/E219K^-RFP, ATG9^KO^/p97^R154C^-RFP and ATG9^KO^/p97^E219K^-RFP, and found that the rescue originally reported in Arhzaouy *et al*. [1] indeed required the presence of both, the R154C and the E219K p97 point mutations. Expression of either R154C or E219K p97 alone was not able to rescue the ATG9^KO^ phenotype (Fig 10). In conclusion, the model of p97 and ATG9 interaction and mutual inhibition as presented in figure 9 of Arhzaouy *et al*. [1] retains its validity, however, the observed effects were due to p97 harboring both point mutations.

**Fig 10:**
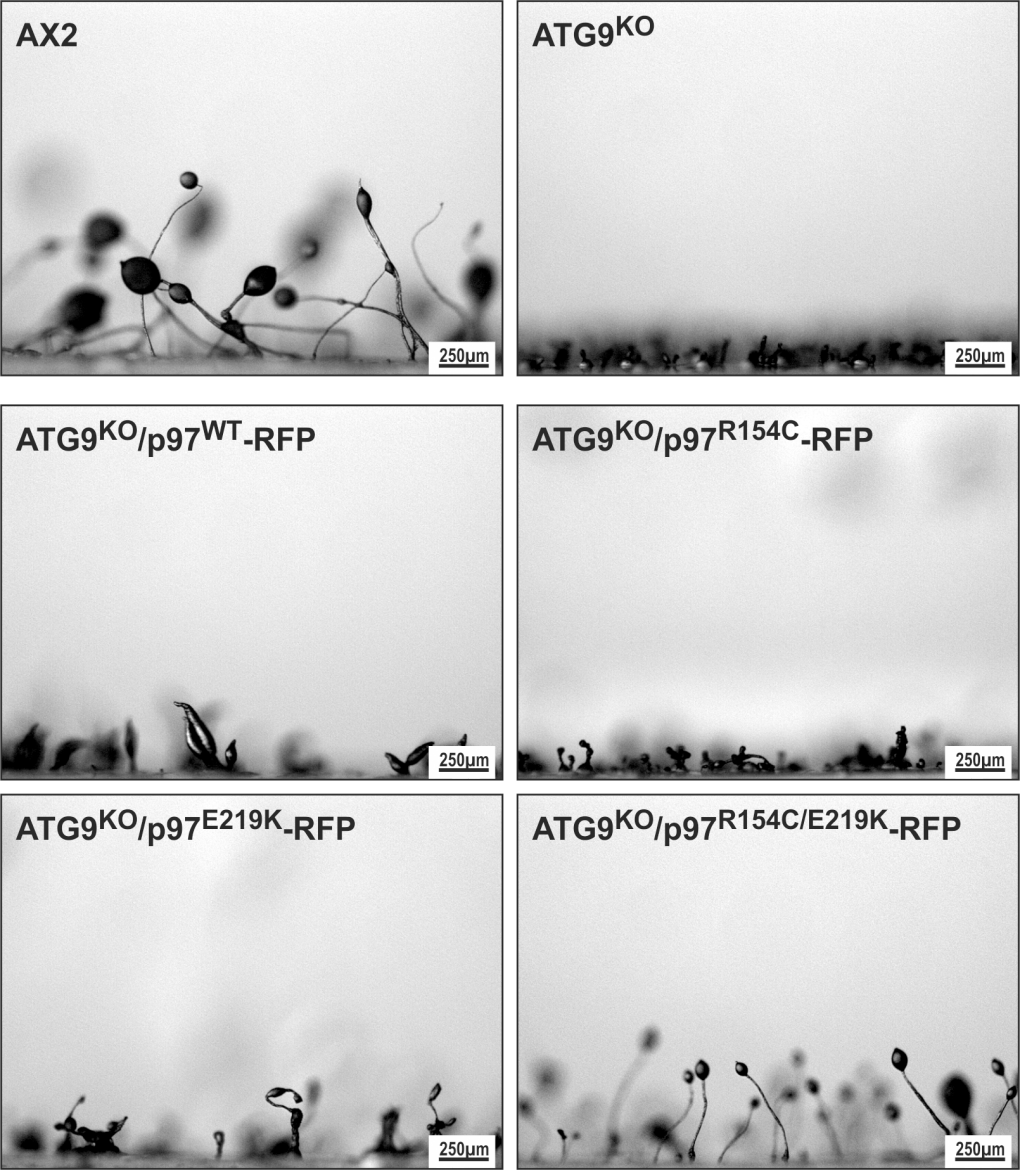
Analysis of fruiting body formation in the *D*. *discoideum* strains AX2 (wild-type), ATG9^KO^ and ATG9^KO^ cells ectopically expressing either wild-type, R154C, E219K or R154C/E219K p97 protein variants fused to RFP. Fruiting body formation, i.e. the formation of a stalk in conjunction with a clear spore head, was completely lost in ATG9^KO^ and almost completely lost in ATG9^KO^ cells that ectopically expressed either wild-type, R154C or E219K single-mutant p97. In contrast, ectopic expression of p97^R154C/E219K^-RFP rescued fruiting body formation in the ATG9^KO^ cells as originally shown in Arhzaouy *et al*. [1].

Fig 10, “Analysis of fruiting body formation in the *D*. *discoideum* strains AX2 (wild-type), ATG9^KO^ and ATG9^KO^ cells ectopically expressing either wild-type, R154C, E219K or R154C/E219K p97 protein variants fused to RFP,” does not appear in the original article. Please view Fig 10 here.
