# Supplementary figures and images for: Heteromeric p97/p97R155C Complexes Induce Dominant Negative Changes in Wild-Type and Autophagy 9-Deficient Dictyostelium strains
Source: PLoS One. 2012 Oct 3;7(10):e46879. doi: 10.1371/journal.pone.0046879 (PMC3463532; doi:10.1371/journal.pone.0046879)

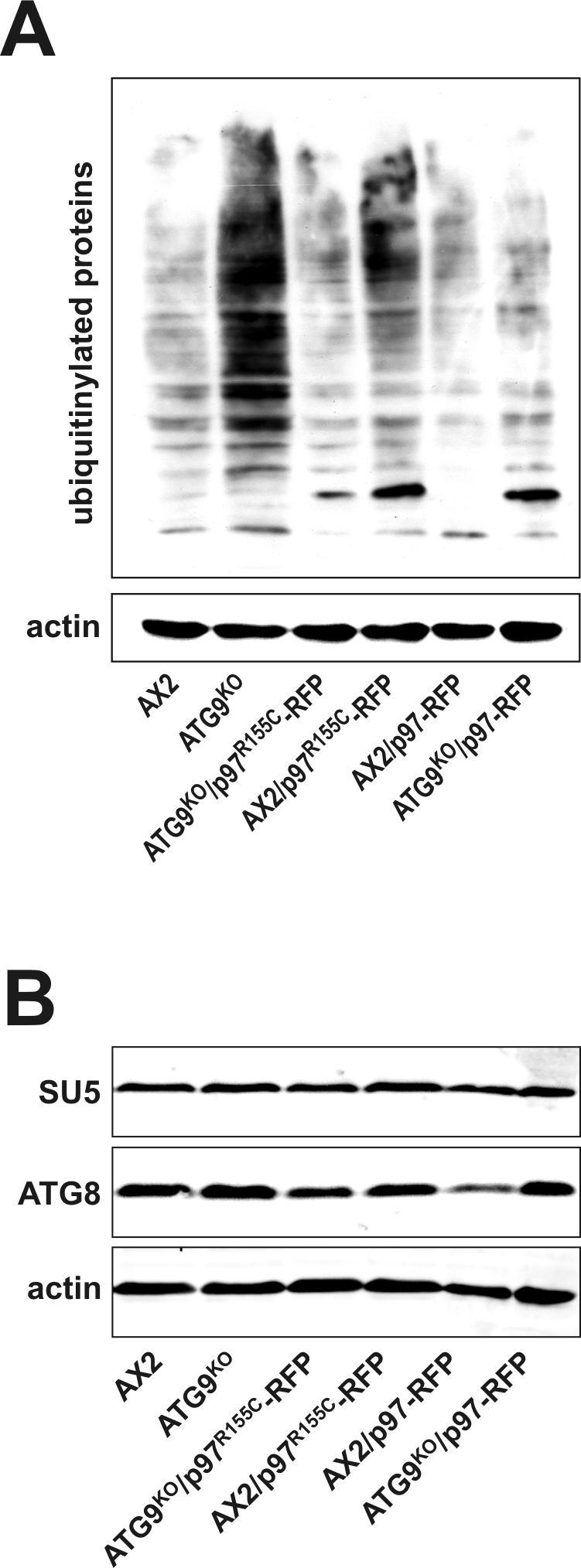

Supplement: Figure S1 — Levels of ubiquitinylated proteins, SU5, and ATG8(LC3). For detection and quantitation of ubiquitin, SU5, ATG8, and actin in Western blots the monoclonal antibodies P4D1 (NEB, Germany), proteasomal subunit 5 (SU5) [36], and Act-1-7 [37] as well as the ATG8_6080 polyclonal antibody were used, respectively. (TIF) [file pone.0046879.s001.tif]
